# Inhibitory Effects of Ferrihydrite on a Thermophilic Methanogenic Community

**DOI:** 10.1264/jsme2.ME14026

**Published:** 2014-05-23

**Authors:** Chihaya Yamada, Souichiro Kato, Yoshiyuki Ueno, Masaharu Ishii, Yasuo Igarashi

**Affiliations:** 1 Department of Biotechnology, Graduate School of Agricultural and Life Sciences, The University of Tokyo, Yayoi 1–1–1, Bunkyo-ku, Tokyo 113–8657, Japan; 2 Bioproduction Research Institute, National Institute of Advanced Industrial Science and Technology, Tsukisamu-Higashi 2–17–2–1, Toyohira, Sapporo, Hokkaido 062–8517, Japan; 3 Division of Applied Bioscience, Graduate School of Agriculture, Hokkaido University, Kita-9 Nishi-9, Kita-ku, Sapporo, Hokkaido 060–8589, Japan; 4 Research Center for Advanced Science and Technology, The University of Tokyo, Komaba 4–6–1, Meguro-ku, Tokyo 153–8904, Japan; 5 Kajima Technical Research Institute, Tobitakyu 2–19–1, Chofu-shi, Tokyo 182–0036, Japan

**Keywords:** inhibition of methanogenesis, thermophilic anaerobic environments, iron reduction, iron oxide, ferrihydrite

## Abstract

The addition of ferrihydrite to methanogenic microbial communities obtained from a thermophilic anaerobic digester suppressed methanogenesis in a dose-dependent manner. The amount of reducing equivalents consumed by the reduction of iron was significantly smaller than that expected from the decrease in the production of CH_4_, which suggested that competition between iron-reducing microorganisms and methanogens was not the most significant cause for the suppression of methanogenesis. Microbial community analyses revealed that the presence of ferrihydrite markedly affected the bacterial composition, but not the archaeal composition. These results indicate that the presence of ferrihydrite directly and indirectly suppresses thermophilic methanogenesis.

Most CH_4_ produced on the earth is thought to be derived from the microbial degradation of organic matter in anoxic environments (25). Methanogenesis is a complex microbial process that involves catabolic interactions among different groups of microorganisms, including primary and secondary fermenting bacteria, homoacetogenic bacteria, and methanogenic archaea (11, 22). Various environmental stimuli, including temperature, pH, and inhibitory compounds, have been shown to affect the activities of methanogenic microbial communities (3, 7, 26). Of these, the effects of iron minerals on microbial methanogenesis have been extensively investigated (28). Although a few highly crystalline iron oxide species have recently been shown to promote methanogenesis (12), readily reducible, poorly crystalline iron oxide species, such as ferrihydrite, have frequently been reported to exhibit inhibitory effects on microbial methanogenesis in natural and artificial environments (6, 10, 14, 18, 19). Two different mechanisms have been proposed for the inhibitory effects of iron oxides on methanogenesis. The first mechanism is the outcompetition of methanogenic archaea by iron-reducing microorganisms for the preferential consumption of common electron donors, such as acetate and H_2_ (1, 6). However, substrate competition alone cannot fully explain the inhibitory effects of iron oxides in different environmental settings (14, 20, 27). The other proposed mechanism is the direct inhibition of methanogens by iron oxides. Methanogens require a low redox potential (<−200 mV versus the standard hydrogen electrode [SHE]) for growth and are inhibited at high redox potentials in microhabitats, such as those caused by the presence of ferrihydrite (−100 to +100 mV versus SHE) (5, 8, 9, 23). In addition, several species of mesophilic and thermophilic methanogens that are able to reduce iron oxides cease methanogenesis in the presence of readily reducible iron(III) species (5, 27, 30). However, most of the previous studies that examined the inhibitory effects of iron oxides on methanogenesis have only focused on mesophilic environments. Although microbial methanogenesis in thermophilic environments is known to have a marked impact on global carbon and energy cycles in diverse natural environments, the inhibitory effects of iron oxides on methanogenesis in thermophilic microbial communities have not yet been investigated.

In the present study, methanogenic communities obtained from a thermophilic anaerobic digester (21) were cultivated in the absence or presence of various concentrations of ferrihydrite to investigate the effects of ferrihydrite on methanogenesis in a thermophilic microbial community. Twenty milliliters of the collected methanogenic sludge was transferred to 70-mL vials. After replacing the gas phase with nitrogen gas, the vials were anaerobically incubated at 55°C under static conditions. Cultivations were conducted using a fed-batch culture technique: 2 mL of the culture solution was removed daily and replaced with 2 mL of Medium A (24), which contained 20 mM sodium acetate and 0.2% (w/v) yeast extract (Difco, BD, US) as the methanogenic substrates. Cultures were also supplemented with ferrihydrite at 0, 2, 4, 8, or 16 mmol Fe(III) L^−1^ d^−1^. Ferrihydrite was synthesized by neutralizing a 0.4 M of FeCl_3_ solution to pH 7 with 10 M NaOH, as previously described (13). The concentrations of the organic acids and alcohols were determined by high-pressure liquid chromatography as described elsewhere (2). The amounts and compositions of the generated biogas were analyzed using a Handy Manometer and gas chromatograph-mass spectrometer, as described previously (21). Data were statistically analyzed using the paired Student’s *t*-test.

The production of CH_4_ began immediately after the addition of medium to the culture. Some organic acids ( acetate, butyrate, propionate, lactate, and formate) were detected, but did not accumulate under all culture conditions at concentrations exceeding 0.2 mM during the cultivation period. The methanogenic rates gradually increased and became nearly constant after 2 weeks of cultivation. The methanogenic rates of each culture from days 15 to 19 are shown in Fig. 1A. The methanogenic rate of the non-Fe control culture was 7.7 ± 1.2 mmol L^−1^ d^−1^. Since 2 mmol L^−1^ CH_4_ is theoretically produced from the complete degradation of 2 mM acetate, most of the CH_4_ produced was likely to have been derived from organic compounds in the yeast extract. The methanogenic rates in the ferrihydrite-supplemented cultures decreased as the concentrations of ferrihydrite increased. The methanogenic rate of the cultures supplemented with 16 mmol Fe(III) L^−1^ d^−1^ ferrihydrite was less than half of that of the control cultures. These results clearly demonstrated that ferrihydrite inhibited microbial methanogenesis under the thermophilic conditions used here, which is consistent with the findings obtained in diverse mesophilic environments (6, 10, 14, 18, 19).

The reddish-brown ferrihydrite particles became black during cultivation of the methanogenic microbial communities, which was indicative of microbial iron reduction. To confirm this, Fe(II) concentrations in the cultures were measured using the ferrozine method (13), and iron reduction rates were determined during days 15 to 19 (Fig. 1B). The marked reduction of iron was observed in cultures supplemented with 8 or 16 mmol Fe(III) L^−1^ d^−1^ of ferrihydrite, while iron reduction rates were marginal in the other cultures. The lower methanogenic rates in ferrihydrite-supplemented cultures than in the non-Fe control are also shown in units of mmol e^−^ equivalents L^−1^ d^−1^ in Fig. 1B as a comparison. The amounts of reducing equivalents consumed by the reduction of iron were only 24% and 27% of that expected from the observed decrease in the production of CH_4_ in the cultures supplemented with 8 and 16 mmol Fe(III) L^−1^ d^−1^ of ferrihydrite, respectively. These results suggested that direct competition between iron-reducing microorganisms and methanogens for common electron donors such as H_2_ and acetate had a slight effect, but was not the major causal factor underlying the ferrihydrite-dependent inhibition of methanogenesis.

Compositional changes in microbial communities in the methanogenic cultures supplemented with 0, 8, and 16 mmol Fe(III) L^−1^ d^−1^ of ferrihydrite were assessed using terminal restriction fragment-length polymorphism (T-RFLP) targeting archaeal and bacterial 16S rRNA genes, as previously described (2, 21). Genomic DNA was extracted from culture samples using a direct lysis protocol involving bead beating (16). In iron oxide-supplemented cultures, iron oxide particles were dissolved with oxalic acid treatments (final concentration of ammonium oxalate, 28 g L^−1^ and oxalic acid, 15 g L^−1^) prior to DNA extraction. Only T-RFs with relative peak intensities of >20% of the total intensity in at least one experimental data set were selected as the predominant T-RFs. Sequencing analysis of the clone libraries of archaeal and bacterial 16S rRNA genes was also performed to identify the origins of the major T-RFs, as described elsewhere (2, 21).

Archaeal T-RFLP analysis clearly demonstrated that two T-RF types were predominant in all examined culture conditions (Figs. 2A and S1A–C). The origins of the two major T-RFs were identified as the aceticlastic methanogen *Methanosarcina thermophila* and hydrogenotrophic methanogen *Methanothermobacter thermautotrophicus* by archaeal clone library analysis (Table S1). The predominant archaeal phylotypes in the cultures supplemented with or without ferrihydrite were highly similar (Fig. 2), although the abundance ratio of *Methanosarcina* sp. T-RF with *Methanothermobacter* sp. T-RF became low in the ferrihydrite-supplemented cultures during the early stages of cultivation (Fig. S1A–C). Although ferrihydrite may have directly inhibited the methanogenic archaea (especially *Methanosarcina* sp. during the initial stage of incubation), archaeal T-RFLP analysis revealed that this may not have been the major causal factor for the suppression of methanogenesis in the present study. This phenomenon is consistent with the findings of a previous study in which the direct inhibitory effects of ferrihydrite on methanogens were alleviated by a rapid reduction in ferrihydrite by iron-reducing microorganisms (27).

In the bacterial T-RFLP analysis, four T-RF types were detected as the major peaks (Figs. 2B and S1D–F). The origins of these major T-RFs were identified as the products of the 16S rRNA genes of *Coprothermobacter proteolyticus*, *Anaerobaculum mobile*, *Defluviitoga tunisiensis*, and uncultured *Firmicutes* bacterium by bacterial clone library analysis (Table S1). The T-RF identified to be the sequence of uncultured *Firmicutes* bacterium only dominated in the day 0 samples and its relative abundance decreased with an increase in the time of incubation, which indicated that this bacterium may be not functionally significant under the present experimental conditions. In the non-Fe control cultures, the relative abundances of the three other major bacterial T-RFs dominated in the bacterial T-RF assemblages at the end of the cultivation experiments. *C. proteolyticus*, *A. mobile*, and *D. tunisiensis* are obligately anaerobic, fermentative bacteria that utilize various carbohydrate and proteinaceous compounds for growth and also generate acetate and H_2_/CO_2_ as major fermentative products, but have different substrate utilization spectra (4, 15, 17). In the control cultures, these three bacterial species may have contributed to the degradation of organic compounds in the yeast extract to produce acetate and H_2_/CO_2_, which could then be converted into CH_4_ by the methanogenic archaea. In contrast, only the T-RF identified to be the sequence of *C. proteolyticus* was dominant in the ferrihydrite-supplemented cultures. One possible explanation for the difference between the control and ferrihydrite-supplemented cultures is that *C. proteolyticus* has the ability to acquire energy via the reduction of ferrihydrite. This is supported by the findings of a previous study in which *C. proteolyticus* appeared to have the ability to utilize anodic electrodes as an electron acceptor (29). Another explanation may be that *A. mobile*- and *D. tunisiensis*- like bacteria are more sensitive to the toxic effects of ferrihydrite and/or its reduced products. Based on the complementary nature of the carbohydrate and amino acid utilization profiles of *C. proteolyticus*, *A. mobile*, and *D. tunisiensis*, and the extremely low bacterial phylotype diversities in the ferrihydrite-supplemented cultures, it is conceivable that the selective predominance of specific fermentative bacteria is a potential mechanism responsible for the observed inhibition of methanogenesis in the presence of ferrihydrite. The reduced diversity of fermentative bacteria may result in the inefficient degradation of mixtures of complex organic matters, such as those found in yeast extract, which may have resulted in the inadequate supply of potential substrates for methanogenesis. To the best of our knowledge, such an indirect inhibitory effect of ferrihydrite on methanogenesis associated with a decrease in the diversity of fermentative bacteria has not yet been reported. Further investigations using other thermophilic (and mesophilic) environments are needed to justify this interpretation.

We demonstrated here that ferrihydrite inhibited microbial methanogenesis under thermophilic conditions, as previously observed in mesophilic environments. Our results indicate that the existence of readily-reducible iron minerals affected the functional microbial components and populations in the thermophilic methanogenic community and also inhibited methanogenesis through several potential chemical and biological interactions, namely, the diversion of electron flow from methanogenesis to the reduction of ferrihydrite, increased redox potential of the environments, and inadequate supply of methanogenic substrates due to a decrease in the diversity of fermentative bacteria.

In this study, the nucleotide sequence data of the clone library analyses have been submitted to GenBank under accession numbers: bacterial sequences, AB908264–AB908267; archaeal sequences, AB908268 and AB908269.

## Supplementary Information



## Figures and Tables

**Fig. 1:**
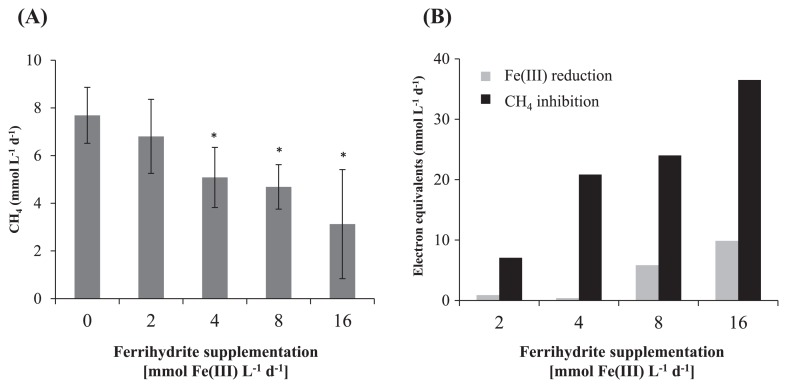
(A) The methanogenic rates of the thermophilic microbial communities in the presence or absence of ferrihydrite during days 15 to 19. (B) Comparison of electron equivalents associated with Fe(III) reduction rates and the decrease in methanogenic rates in the cultures of thermophilic microbial communities. The decrease in the methanogenic rates in ferrihydrite-supplemented cultures subtracted from the non-Fe control was used to calculate the mmol e^−^ equivalents L^−1^ d^−1^. Data are presented as the means of four or five time points, and error bars represent standard deviations. Asterisks represent a significant difference (*P*<0.05) from the Non-Fe control culture.

**Fig. 2:**
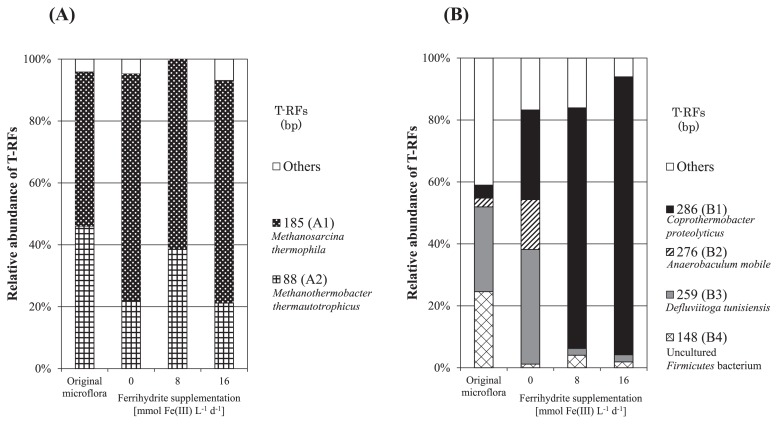
Archaeal (A) and bacterial (B) community structures evaluated by the T-RFLP analysis for the original microflora (day 0) and thermophilic methanogenic cultures in the presence and absence of ferrihydrite (day 19). The length of each T-RF and the corresponding microorganisms identified by clone library analysis are presented.
