# Supplementary material for: Inhibitory Effects of Ferrihydrite on a Thermophilic Methanogenic Community
Source: Microbes Environ. 2014 May 23;29(2):227–30. doi: 10.1264/jsme2.ME14026 (PMC4103531; doi:10.1264/jsme2.ME14026)
Supplement: Supplementary file 1 [file 29_227_s1.pdf]

Supplemental Table 1 **Identification of T-RFs by bacterial and archaeal 16S rRNA gene clone library analyses**

	Characteristic T-RFs (bp)	Phylogenetic group (Phylum or Order)	Closest relative microorganisms (Accession No.)	Similarity (%)
B1	286	*Firmicutes*	*Coprothermobacter proteolyticus* (CP001145)	99
B2	276	*Synergistetes*	*Anaerobaculum mobile* (NR102954)	100
B3	259	*Thermotogae*	*Defluviitoga tunisiensis* (FR850164)	100
B4	148	*Firmicutes*	*Moorella glycerini* (NR029198)	86
A1	185	*Methanosarcinales*	*Methanosarcina thermophila* (JQ346758)	100
A2	88	*Methanobacteriales*	*Methanothermobacter thermautotrophicus* (NR074260)	100

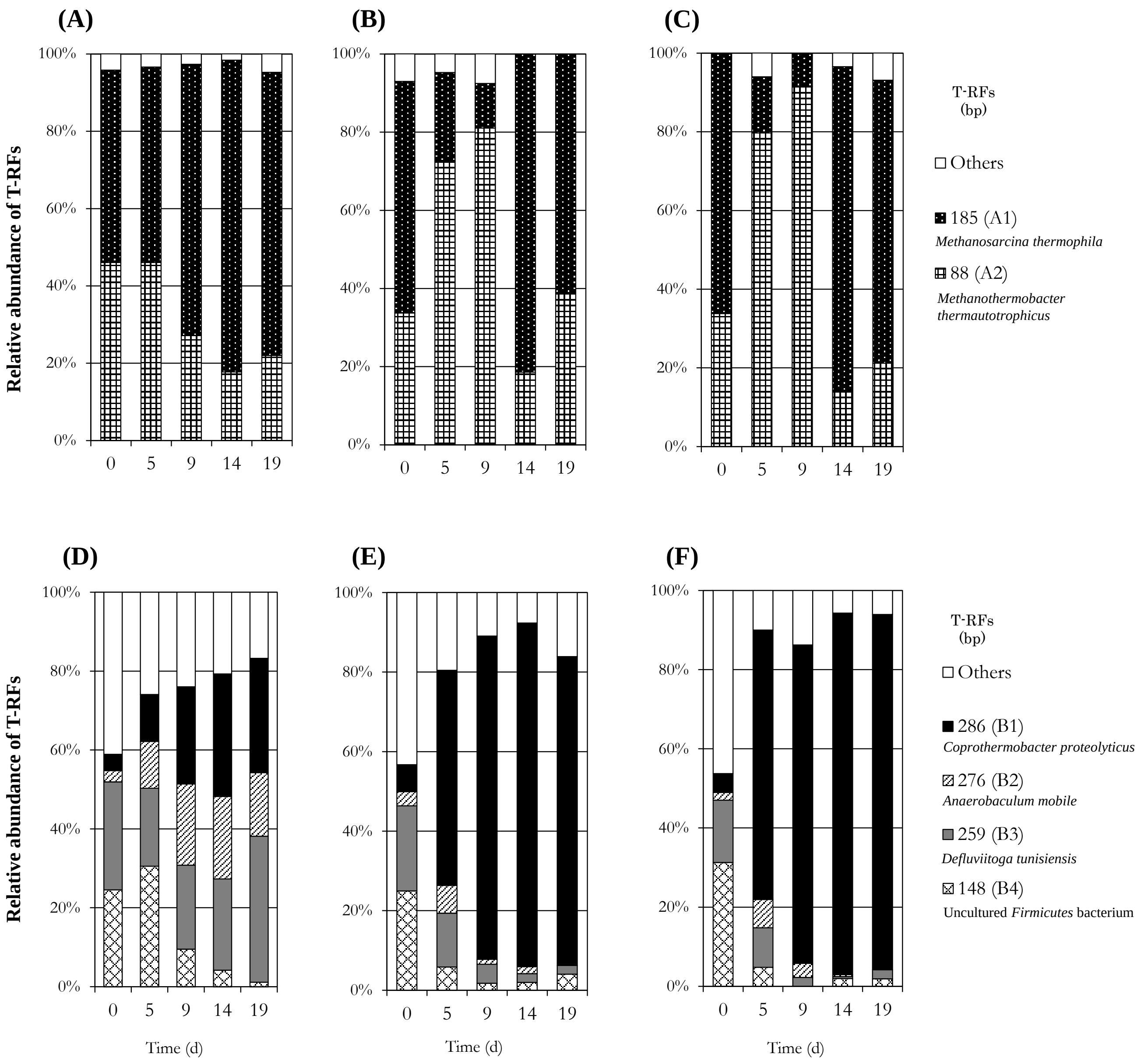

Supplemental Figure 1

Microbial T-RF relative abundances were evaluated by T-RFLP analysis for cultures supplemented with yeast extract, acetate and 0 (A), 8 (B) or 16 mmol Fe(III) L^{-1} day^{-1} (C) ferrihydrite for archaeal communities and 0 (D), 8 (E) or 16 mmol Fe(III) L^{-1} day^{-1} (F) ferrihydrite for bacterial communities.
